# LHC physics dataset for unsupervised New Physics detection at 40 MHz

**DOI:** 10.1038/s41597-022-01187-8

**Published:** 2022-03-29

**Authors:** Ekaterina Govorkova, Ema Puljak, Thea Aarrestad, Maurizio Pierini, Kinga Anna Woźniak, Jennifer Ngadiuba

**Affiliations:** 1 European Organization for Nuclear Research (CERN), CH-1211 Geneva 23, Switzerland; 2 Fermi National Accelerator Laboratory, Batavia, IL 60510 USA; 3 Present Address: University of Vienna, Vienna, Austria; 4 Present Address: California Institute of Technology, Pasadena, CA 91125 USA

**Keywords:** Experimental particle physics, Research data

## Abstract

In the particle detectors at the Large Hadron Collider, hundreds of millions of proton-proton collisions are produced every second. If one could store the whole data stream produced in these collisions, tens of terabytes of data would be written to disk every second. The general-purpose experiments ATLAS and CMS reduce this overwhelming data volume to a sustainable level, by deciding in real-time whether each collision event should be kept for further analysis or be discarded. We introduce a dataset of proton collision events that emulates a typical data stream collected by such a real-time processing system, pre-filtered by requiring the presence of at least one electron or muon. This dataset could be used to develop novel event selection strategies and assess their sensitivity to new phenomena. In particular, we intend to stimulate a community-based effort towards the design of novel algorithms for performing unsupervised new physics detection, customized to fit the bandwidth, latency and computational resource constraints of the real-time event selection system of a typical particle detector.

## Abstract (summary)

**Table:** 

Measurement(s)	Simulations of LHC collisions in real-time processing data format
Technology Type(s)	PYTHIA, DELPHES, private python code
Factor Type(s)	phi • eta • pT

## Background & Summary

The proton bunches of the CERN Large Hadron Collider (LHC) cross paths ${\mathcal{O}}(10)$ million times per second in each of the experimental halls, possibly generating a *collision event* each time. In such an event multiple proton pairs may collide, possibly producing thousands of particles to be detected by the detectors located in each experimental hall. Detector sensors record the flow of emerging particles in the form of electronic signals. For the so-called general purpose detectors (ATLAS^1^ and CMS^2^), this globally amounts to ${\mathcal{O}}(1\,{\rm{MB}})$ of information. The resulting data throughput of ${\mathcal{O}}(10)$ TB/s is too large to be recorded. This is why these two detectors process data in real-time to select a small fraction of them (about 1000/s), compatible with downstream computing resources. This strategy was effective in providing the data needed to discover the Higgs boson^3,4^.

**Fig. 1:**
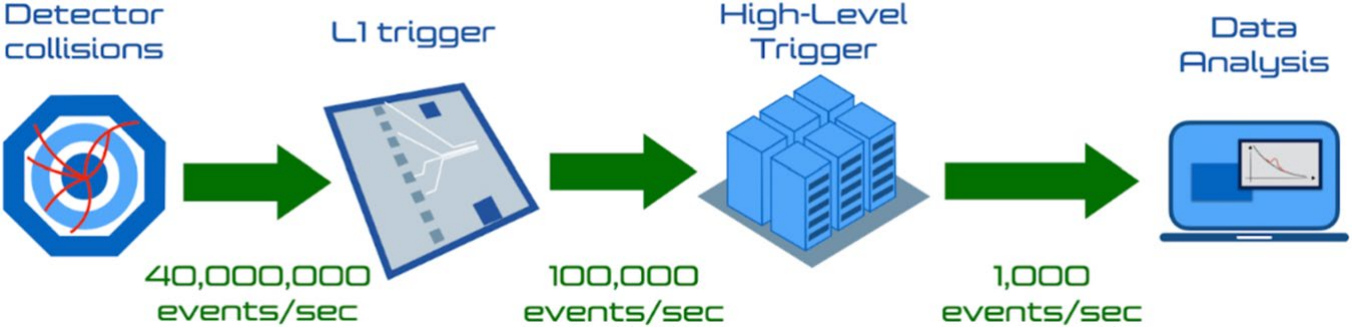
The real-time data processing flow of the ATLAS and CMS experiments: ${\mathcal{O}}(10)$ M collisions are produced every second and processed by the hardware-based event selection system, consisting of algorithms implemented as logic circuits on custom electronic boards. Of these events, 100k events/s are accepted and passed to the second selection stage, the HLT, which selects about 1000 events/s for offline physics studies.

This filtering system, usually referred to as the *trigger*, consists of a two-stage selection, as illustrated in Fig. 1. With the recent upgrade of their data acquisition system, the two other big detectors, LHCb and ALICE, have been equipped with a novel data processing capability that avoids the need to select events. Instead, their data acquisition follows a real-time data processing approach^5,6^: all the events enter a computer farm, where they are processed in real-time and stored in a reduced-size data format, consisting of high-level information sufficient for data analysis. For this reason, their case goes beyond the scope of this paper, and it is not considered further.

The first trigger stage, the Level-1 trigger (L1T), runs a set of algorithms deployed as logic circuits on custom electronic boards equipped with field-programmable gate arrays (FPGAs). This stage rejects more than 98% of the events, reducing the incoming data stream to 100k events/s. Due to the short time interval between two events (25 ns) and limited buffer capabilities, the entire pipeline of L1T algorithms has to be executed within ${\mathcal{O}}(1)\,\mu {\rm{s}}$. The second stage, called the high-level trigger (HLT), consists of a computer farm processing events on commercial CPUs, running hundreds of complex selection algorithms within ${\mathcal{O}}(100)\,{\rm{ms}}$. The trigger selection algorithms are designed to guarantee a high acceptance rate for the physics processes under study.

When designing searches for unobserved physics phenomena, one typically considers specific theory-motivated scenarios. This *supervised* strategy has been proven successful when dealing with strongly theoretically motivated searches, e.g., the Higgs boson discovery^3,4^. However, this approach might become a limiting factor in the absence of strong theoretical guidance. The ATLAS and CMS trigger systems could be discarding interesting events, limiting the possibility of new physics discoveries.

Therefore, recent work has investigated *unsupervised* and *semi-supervised* approaches to data selection and analysis, focusing on anomaly detection (AD) strategies based on deep learning (DL) algorithms. These studies aim to learn a metric directly from the LHC data, with the capability of ranking the events by typicality. One could select a sample enriched with anomalies, possibly due to new-physics processes, by selecting all the events in the tail of the distribution of such a metric. Extensive reviews of several proposed methods are given in Refs. ^7,8^ and references therein.

This effort, mainly targeting offline data analysis, should be paired with a similar effort to integrate AD algorithms in the trigger system of the LHC experiments, possibly already at the L1T. There, it would be possible to present an unbiased dataset to the AD algorithm, before discarding any event^9,10^. One could then collect rare event topologies in a special data stream, similar to what was done at CMS with the *exotica hotline*^11,12^ during the first year of data taking at the LHC. By studying these events, one could formulate new theoretical models of new physics phenomena, that could be tested in future data-taking campaigns.

While the focus so far has been on the HLT, this strategy would be more effective if deployed in the L1T, before any selection bias is introduced. Each L1T event has to be processed within a few microseconds and therefore the trigger decision is taken by algorithms hard-coded in the hardware electronics as logic circuits. Algorithm complexity, and possibly accuracy, could be enhanced by deploying DL algorithms in the L1T FPGAs. To do so, the hls4ml library^13–15^ was introduced as a tool to translate a given DL model into an electronic circuit. Commercial AI-on-FPGA software libraries are mainly designed to exploit the FPGA as an acceleration device, to support a commercial CPU in its network-inference computation. This implies the serial reuse of specific computing for components for many tasks. On one hand, this allows one to execute large networks (as in computing vision applications). On the other hand, the serial nature of the calculation and the consequent data transfer implies a latency time that exceeds by orders of magnitude what can be tolerated in a L1T system at the LHC. By integrating the full network on the FPGA, hls4ml prioritizes inference speed, avoiding data transfer in sequential steps. The consequent latency reduction comes at a cost in terms of resource utilization. This is why hls4ml is ideal for small networks with ${\mathcal{O}}(100\,{\rm{ns}})$ latency.

Given this, all the ingredients are there to make it possible for the community to design an optimal AD strategy for the L1T system. In order to stimulate this effort as a community-based initiative, similarly to what was done with the LHC Olympics^7^ and the Dark Machine data challenge^8^, we present a new dataset designed to the kind of data stream that one could handle at the last stage of the ATLAS and CMS L1T systems.

## Methods

### Physics content of the dataset

The proton-proton collisions taking place at the LHC can lead to the production and observation of many different processes predicted by the Standard Model (SM) of particle physics^16–18^. A brief summary of the SM particle content can be found in Refs. ^19,20^. The rate at which each of these processes occur can be calculated within the SM mathematical framework and then validated by the measurements performed by the experiments (summary of the CMS cross section measurements is available here). In this paper, we focus on events containing electrons (*e*) and muons (*μ*), light particles that, together with taus (*τ*) and their neutrino partners, form the three lepton families. In principle, we could have considered a dataset with no filter. While this would certainly be a more realistic representation of an unbiased L1T stream, generating such a dataset requires computing resources beyond our capabilities. Instead, we decided to use the lepton filter and make the dataset simulation tractable.

Within the limited size of a typical LHC detector, electrons and muons are stable particles, i.e., they do not decay in the detector and they are directly observed while crossing the detector material. On the contrary, *τ* leptons are much heavier, and hence much more unstable, than electrons and muons. They quickly decay into other stable particles. In a fraction of these decays, *e* and *μ* are produced. At the LHC, the most abundant source of high-energy leptons is the production of *W* and *Z* bosons^21^ which are among the heaviest SM particles with a mass of ~80 and ~90 proton masses, respectively. Once produced, they quickly decay into other particles. In a small fraction of cases, these particles are leptons. *W* and *Z* bosons are mainly produced directly in proton collisions. A sizable fraction of *W* bosons originate from the decay of top quarks (*t*) and anti-quarks ($\bar{t}$). The top quark being heavy and highly unstable, quickly decays into a *W* boson and a bottom quark, giving rise to signatures with only collimated sprays of hadrons called *jets* or with one *e*, *μ*, or *τ*, a neutrino and multiple jets. Leptons can originate from more rare *W* and *Z* production, such as from the decay of Higgs bosons or multi-boson production. Given the small production probability of these processes, we ignore them in this study.

As predicted by quantum chromodynamics (QCD)^22^, most of the LHC collisions result in the production of light quarks (up, down, charm, strange, and bottom) and gluons. As these quarks and gluons have a net colour charge and cannot exist freely due to colour-confinement, they are not directly observed. Instead, they come together to form colour-neutral hadrons, in a process called hadronisation that leads to jets. Sometimes, leptons can be produced inside jets, typically from the decay of unstable hadrons. Since QCD multijet production is by far the most abundant process occurring in LHC collisions, the production of leptons inside jets becomes relevant. Therefore, this contribution is sizable and taken into account.

### Dataset

The processes listed above are the main contributors to an *e* or *μ* data stream, i.e., the set of collision events selected for including an *e* or *μ* with energy above a defined threshold. One of the datasets presented in this paper consists of the simulation of such a stream. In addition, benchmark examples of new lepton-production processes are given. These processes consist of the production of hypothetical, but still unobserved particles. They serve as examples of data anomalies that could be used to validate the performance of an AD algorithm. Details on these processes can be found in Refs. ^9,10^.

Moreover, we published a *blackbox* dataset containing a mixture of SM processes and a *secret* signal process. Events in this dataset are uniquely labeled by an event number, which allows us to link each event to the corresponding *ground-truth* dataset, containing a set of 0 (for SM events) and 1 (for new physics events) bits. The ground truth dataset is stored on a private cloud storage area at CERN. By not publishing this dataset, we can assure that the distributed *blackbox* is unlabeled for external developers. It is intended to be used to independently validate the performance of AD algorithms developed based on this dataset.

**Fig. 2:**
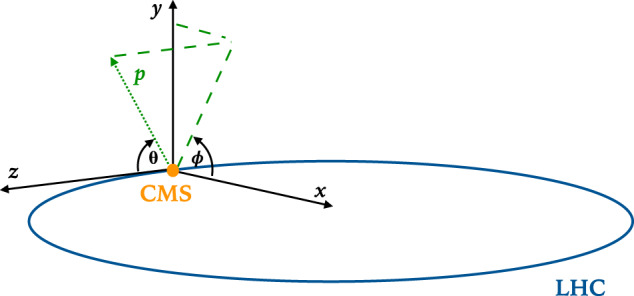
The reference system used to describe the momentum coordinates of the particles in the dataset.

To describe the data, we use a right-handed Cartesian coordinate system with the *z* axis oriented along the beam axis, the *x* axis toward the center of the LHC, and the *y* axis oriented upward as shown in Fig. 2. The *x* and *y* axes define the transverse plane, while the *z* axis identifies the longitudinal direction. The azimuth angle *ϕ* is computed with respect to the *x* axis. Its value is given in radians, in the [−*π*, *π*] range. The polar angle *θ* is measured from the positive *z* axis and is used to compute the pseudorapidity $\eta =-{\rm{\log }}({\rm{\tan }}(\theta /2))$. The transverse momentum (*p*_*T*_) is the projection of the particle momentum on the (*x*, *y*) plane. We use natural units such that $c=\hbar =1$ and we express energy in units of electronvolt (eV) and its prefix multipliers.

Each event is represented by a list of four-momenta for high-level reconstructed objects: muons, electrons, and jets. In order to emulate the limited bandwidth of a typical L1T system, we consider only the first 4 muons, 4 electrons, and 10 jets in the event, selected after ordering the candidates by decreasing *p*_*T*_. If an event contains fewer particles, the event is zero-padded to preserve the size of the input, as is done in realistic L1T systems. Each particle is represented by its *p*_*T*_, *η*, and *ϕ* values. In addition, we consider the absolute value and *ϕ* coordinates of the missing transverse energy (MET), defined as the vector equal and opposite to the vectorial sum of the transverse momenta of all the reconstructed particles in the event.

Since the current trigger system was designed without having this application in mind, we assumed that one would have to keep the deployment as minimally intrusive as possible. This is why the event data format is built out of quantities available at the last stage of the trigger (having in mind the CMS design), under the assumption that any AI-based algorithm running on this information would be executed as the last step in the L1-trigger chain of computations.

Once generated, events are filtered using a custom selection algorithm, coded in Python. This filter requires a reconstructed electron or a muon to have *p*_*T*_ > 23 GeV, within |*η*| < 3 and |*η*| < 2.1 respectively. For the events that pass the requirement, up to ten jets with *p*_*T*_ > 15 GeV within |*η*| < 4 are included in the event, together with up to four muons with |*η*| < 2.1 and *p*_*T*_ > 3 GeV, up to four electrons with |*η*| < 3 and *p*_*T*_ > 3 GeV, and the missing transverse energy, as defined earlier. Given these requirements, the four SM processes listed below provide a realistic approximation of a L1T data stream. While the dataset has a L1-like format, the physics content is not that of an unbiased trigger (zero bias data), because of this single-lepton pre-filtering. As explained in Refs. ^9,23^, this cut was introduced for practical reasons, to make the dataset manageable given our limited computing resources.

The following SM processes are relevant to this study (charge conjugation is implicit):

- Inclusive *W* boson production, where the *W* boson decays to a charged lepton (ℓ) and a neutrino (*v*), (59.2% of the dataset). The lepton could be a *e*, *μ*, or *τ* lepton.
- Inclusive *Z* boson production, with $Z\to \ell \ell (\ell =e,\mu ,\tau )$ (6.7% of the dataset),

- $t\bar{t}$ production (0.3% of the dataset), and

- QCD multijet production (33.8% of the dataset).
  The relative contribution to the dataset listed in parenthesis take into account the production cross section (related to the probability of generating a certain process in an LHC collision) and the fraction of events accepted by the event selection described above. These four samples are mixed to form a realistic data stream populated by known SM processes (collectively referred to as *background*), and is provided in Ref. ^24^. An AD algorithm can thus be trained on this sample to learn the underlying structure of the background to identify a new physics signature (the *signal*) as an outlier in the distribution of the learned metrics.
  To study the performance of AD algorithms, four signal datasets are provided:
- A leptoquark (LQ) with an 80 GeV mass, decaying to a *b* quark and a *τ* lepton^25^,
- A neutral scalar boson (*A*) with a 50 GeV mass, decaying to two off-shell *Z* bosons, each forced to decay to two leptons: *A* → 4ℓ^26^,
- A scalar boson with a 60 GeV mass, decaying to two tau leptons: *h*^0^ → *ττ*^27^,
- A charged scalar boson with a 60 GeV mass, decaying to a tau lepton and a neutrino: *h*^±^ → *τv*^28^.

These samples are generated using the same code and workflow as the SM events.

In total, the SM cocktail dataset consists of 8,209,492 events, of which 4 million are used to define the training dataset^24^. The rest are mixed with events from the secret new physics process, to generate the *blackbox* dataset^29^. Together with the particle momenta, this dataset includes the event numbers needed to match each event to its ground-truth bit. The signal-benchmark samples in Refs. ^25–28^ amount to a 1,848,068 events in total. These data are available to test specific algorithms before running them on the blackbox.

## Data Records

The publication consists of six data records: one record containing the mixture of SM processes, four separate records for each of the beyond the Standard Model (BSM) processes listed above and one record containing the *blackbox* data. These are listed in Table 1, together with the total number of events and whether the record is considered to be background, signal or a mixture of the two.

**Table 1:** The names and corresponding Zenodo reference for each dataset, the total number of collision events and the dataset type (S for signal and B for background).

Sample name	Number of events	Type
SM processes^24^	4,000,000	B
*LQ* → *bτ*^25^	340,544	S
$A\to 4\ell$^26^	55,969	S
*h*^0^ → *ττ*^27^	691,283	S
*h*^±^ → *τν*^28^	760,272	S
*blackbox*^29^	4,210,492	S + B

The datasets are revised versions of those utilized in Refs. ^9,10^ and already published on Zenodo^30–37^. They differ from each other in data format, and the inclusion of a *blackbox* dataset containing secret new physics events. The benchmark new physics datasets have the same physics content as the original datasets, but the included events were generated specifically for this paper. The data records are published on Zenodo^24–29^.

The data records are provided in Hierarchical Data Format version 5 (HDF5), and contain 3 datasets: “Particles”, “Particles_Classes” and “Particles_Names”. The dataset “Particles” has a shape (N, 19, 4), where N is the number of events listed for each sample in Table 1. The second index runs over the different physics objects in the events: MET, 4 electrons, 4 muons, 10 jets. Its cardinality (19) is the maximum number of objects per event. If fewer objects are present, the event is zero padded in such a way that the 1st, 5th, and 9th positions correspond to the highest-*p*_*T*_ electron, muon, and jet, respectively. The last index (with cardinality 4) runs over the three features describing each physics object and a particle type index, which is equal to 1, 2, 3 and 4 for MET, electron, muon and jet, respectively. The information of which particle kind corresponds to which index value is contained in the “Particle_Classes” dataset, in the form of a single-entry array of strings. Zero padding is done inclusively, e.g. for zero-padded particles the particle type index is set to zero. The features are ordered as described in the “Particles_Names” dataset: *p*_*T*_, *η*, *ϕ*. The *blackbox* sample includes an additional dataset (“EvtId”) with dimension (N), containing an event ID which allows us to match each event to its ground truth (signal or background).

## Technical Validation

**Fig. 3:**
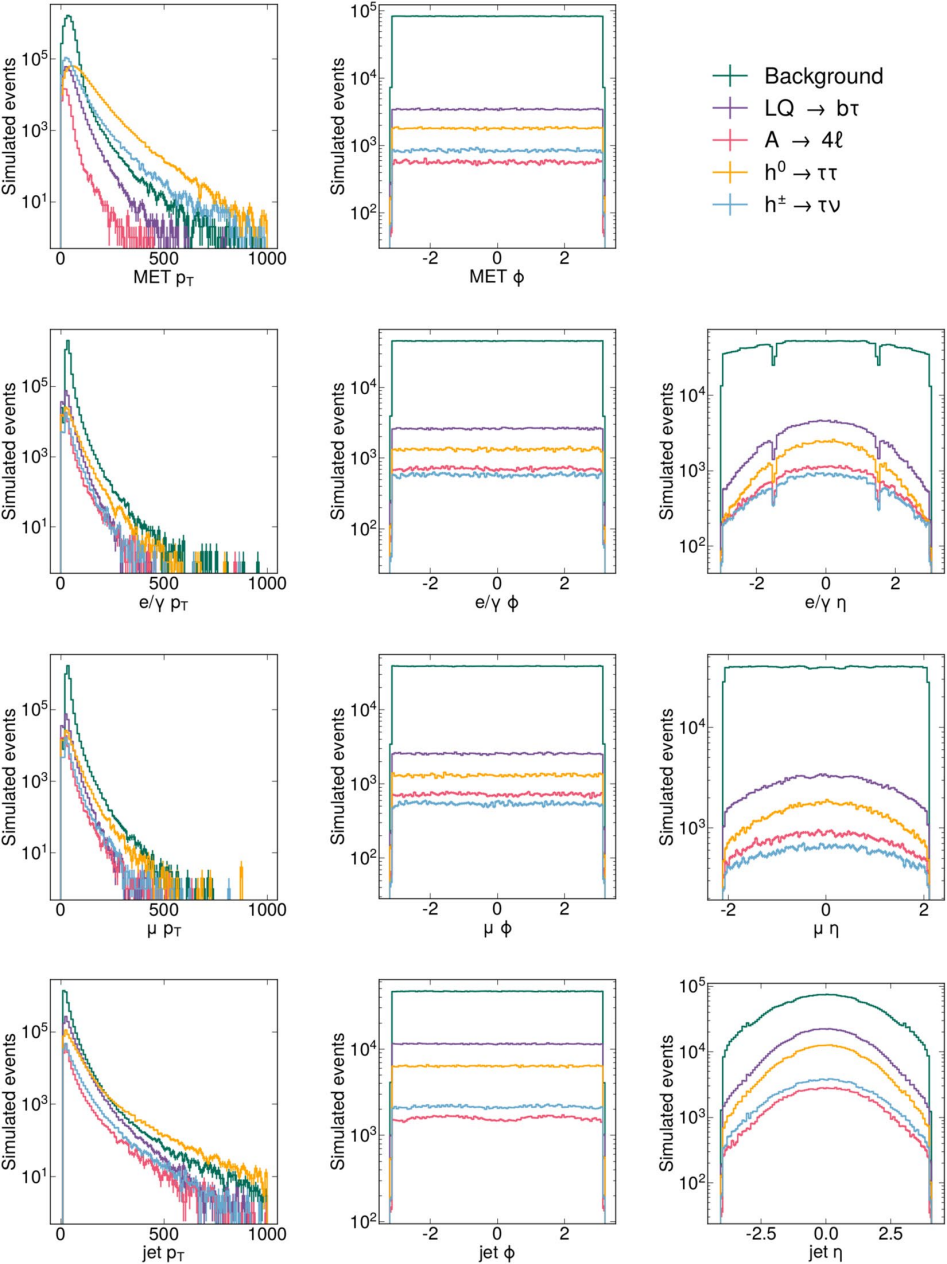
Distribution of the *p*_*T*_ (left), *ϕ* (center) and *η* (right) coordinates of the physics objects entering the dataset, for missing transverse energy, MET (top row), electrons (second row), muons (third row) and jets (bottom row).

The distributions of the features for the SM processes and for the chosen BSM models are shown in Fig. 3. All expected features are observed, e.g., the detector *ϕ* symmetry, the detection inefficiency in *η* in the transition regions between detector components, and the different *p*_*T*_ distributions for the different processes.

## Usage Notes

The dataset publication is accompanied by a Python-based software package, providing examples of how to read the data, train an AD algorithm with them, apply it to the *blackbox* dataset and publish the result (the unique identifier of the 1000 most anomalous events according to some given AD metric)^38^. We aim at challenging the interested data scientists within and outside of the LHC community to design L1T-friendly AD algorithms, and to submit their list of the 1000 most anomalous events to a specific GitHub repository^39^. The final goal is to document these algorithms in a dedicated publication and to preserve the benchmark data for future studies.

## Data Availability

Data are generated using PYTHIA 8.240^40^, setting the collision energy at 13 TeV. Unless otherwise specified, all parameters were fixed to their default values. We set the beam parameters to produce proton-proton collisions at 13 TeV Beams:idA = 2212 ! first beam, p = 2212, pbar = −2212 \\ Beams:idB = 2212 ! second beam, p = 2212, pbar = −2212 \\ Beams:eCM = 13000 .! CM energy of collision \\ while the rest of the card is configured specifically for each process, as indicated in the PYTHIA manual^40^. For example, *W* → ℓ*v* decays are generated with the settings: WeakSingleBoson:ffbar2W = on! switch on W production mode 24::onMode = off! switch off any W decay 24:onIfAny = 11 13 15! switch on W- > lv decays. The signal-specific parameters for the four benchmark signal models are set as follows: • For *A* → 4ℓ: set the Higgs mass to 50 GeV, force the decay to *Z*^*^*Z*^*^ final states, and force *Z*^*^ → ℓℓ decays (ℓ = *e*,*μ*,*τ*). • For *LQ* → *bτ*: set the *LQ* mass to 80 GeV and force its decays to a *b* quark and a *τ* lepton. • For *h*^0^ → *ττ*: set the Higgs boson mass to 60 GeV and switch off any decay mode other than *ττ*. • For *h*^+^ → *τv*: set the charged Higgs boson mass to 60 GeV and switch off any decay mode other than *τv*. We emulate the detector response with DELPHES 3.3.2^41^, using the default Phase-II CMS detector card. For simplicity, we avoid degrading the detector resolution to account for the coarser nature of L1T event reconstruction. This simplification does not affect the aim of the study, which is not focused on assessing the absolute physics performance but instead on comparing different algorithms and their resource consumption. We include the effect of parasitic proton collisions, sampling the number of collisions according to a Poisson distribution centered at 20. The Delphes outcome is processed by a custom Python macro to store the aforementioned physics content on HDF5 files, which are then published.
